# Study of optimization of process parameters on the wear behaviour of Al7075–aluminium oxide composites using Taguchi approach

**DOI:** 10.1038/s41598-025-05188-6

**Published:** 2025-07-01

**Authors:** Vijaya Kumar R, Venugopal M M, Jaya Christiyan K G, Jagadeesha T, Avilasha B G, Shashidhar L C, Raju K, Subraya Krishna Bhat, Thanuj Kumar M, Kanneganti Jyothishya Brahma Chari, Hemanth Raju T, Udayashankar S

**Affiliations:** 1 Aeronautical Engineering Department, MVJ College of Engineering (Affiliated to VTU), Bangalore, 560067 India; 2 Department of Aeronautical Engineering, Nitte Meenakshi Institute of Technology, Bangalore, 560064 India; 3 Department of Mechanical Engineering, Ramaiah Institute of Technology (Affiliated to VTU), Bangalore, 560054 India; 4 Mechanical Engineering Department, National Institute of Technology, Calicut, Kerala 673601 India; 5 Department of Mechanical Engineering, Dayananda Sagar College of Engineering, Bengaluru, 560078 India; 6 Mechanical Engineering Department, Dayananda Sagar University, Ramanagara, Bengaluru, Karnataka 562112 India; 7 Mechanical Engineering Department, St. Joseph Engineering College, Mangalore, 575028 India; 8 Department of Mechanical and Industrial Engineering, Manipal Institute of Technology, Manipal Academy of Higher Education, Manipal, 576104 India; 9 Department of Robotics and Automation, Rajarajeswari College of Engineering, Bengaluru , 560074 India; 10 Department of Civil Engineering, Koneru Lakshmaiah Education Foundation, Vaddeswaram, Andhra Pradesh 522302 India; 11 Mechanical Engineering Department, New Horizon College of Engineering, Bengaluru, 560103 India; 12 Department of Mechanical Engineering, VTU, Belgaum, Karnataka 590018 India

**Keywords:** Al7075, Aluminum oxide, ANOVA, Load, Taguchi

## Abstract

Aluminium alloy based composites are employed in numerous applications that require outstanding performance due to their superior mechanical characteristics, including higher strength, stiffness, and wear resistance. They are used in engine parts like pistons and connecting rods to improve performance and durability. In this work, Al7075 is employed as the matrix material. Aluminium oxide particulates were chosen as the reinforcing particles. The Al7075–6%Al_2_O_3_ composites were manufactured using the stir casting technology. Scanning electron microscopic instrument was employed to investigate the microstructure of Al7075–6%Al_2_O_3_ composites. The microstructure analysis of Al7075–6%Al_2_O_3_ composites revealed the even dispersion of Al_2_O_3_ particulates throughout the Al7075 matrix. The Pin on disc apparatus was utilized to conduct a wear experiment on Al7075–Al_2_O_3_ composites. Taguchi methodology was employed to optimize the wear process factors of the produced composites for enhanced performance. According to ANOVA outcomes, the most impacting factor was the sliding distance 87.057% then speed 7.165% and lastly load 0.435%. The R-Sq value and R-Sq (adj) value for wear response obtained using Minitab 16 Taguchi software are 95.05% and 92.08% respectively. The delta values for load, speed and sliding distance are 0.668, 2.830 and 10.734 respectively. The results of this demonstrated that the factor that has the greatest impact is sliding distance. The wear response values provided by OA experimental and regression equation are 3.6131 × 10^−3^ mm^3^/m and 3.3062 × 10^−3^ mm^3^/m respectively. A difference of 8.49% between the experimental and Taguchi analysis value gives the maximum permissible difference.

## Introduction

The fabrication of composites involves the mixture of two or more distinct components to form a novel material with superior characteristics. The separate components of these materials are not capable of achieving the properties that are intended to be achieved by these materials alone. Composites comprising metal as a matrix, are a category of advanced materials that can be manufactured by dispersing reinforcing phases into a metal matrix for enhancing the properties of the base material^1^. They are extremely strong materials that possess the desirable properties of both the matrix phase and reinforcement materials to make them perform better than traditional metals or alloys.

Aluminium alloys are used in vehicle manufacturing to lower vehicle weight and improve fuel economy. Aluminium alloy’s lightweight, durable properties lend themselves to various applications including engine blocks, cylinder heads, and body panels^2,3^. Because of the beneficial strength to weight ratio, high-strength aluminum alloys are used in the aerospace sector such as example, fuselages and wings.

Aluminium alloys are widely used in industrial machines and equipment for their strength and weight properties. Because of their corrosion resistance and lightweight features, aluminium alloys are used to construct boat hulls and other structures for the marine world, which helps improve performance and the longevity of structures^4,5^.

Aluminium alloy MMC’s are a class of composites that have aluminium alloys as their matrix material in which various reinforcements are introduced with the purpose of enhancing the characteristics of the base material. The two materials are intended to be used in combination to exploit the advantages of both the aluminium matrix and the reinforcing phase, resulting in improved material properties^6^. Its application directly includes bending of aircraft structural components. They are used in automotive parts like pistons and braking components to improve performance and fuel economy.

Aluminium composites are lightweight, high-strength materials which are suitable for aerospace, automotive and construction fields. Aluminium composites provide good thermal conductivity and electrical conductivity, which is ideal for heat sinks and electrical components. Unlike other materials, aluminium composite materials are easy to cut, bend and install, providing considerable savings on labor costs and construction time^7,8^. They can withstand temperature fluctuations, UV rays and moisture, making them perfect for outbound applications.

Aluminium composites can be cost-prohibitive compared to some traditional materials. Aluminium composites are unsuitable for heavy structural loads, unlike solid aluminium or steel. Certain aluminium composites use cores made of plastic or resin, making recycling more challenging than pure aluminium. When heated, aluminium expands, so its correct installation may be complicated, so that over time it does not get damaged or deformed^9^.

Al7075 alloy is having high strength with good mechanical properties. It is suitable for structural aerospace applications. Its mechanical properties are good for aerospace, automotive and high performance but appropriate handling is necessary to maintain its lower corrosion resistance. Aluminium oxide is a crucial material because of its hardness, stability and available uses. It is a primary ceramic material and also functions in industrial as well as electronic applications. Stir casting is a common method of fabrication for MMC’s in general, but especially when trying to add solid particles into a molten metal matrix^10^. It is a handy method when it comes to producing composites in mass with substantial ease and at economic rates.

Al7075 alloy is extensively employed as a matrix material in Al7075–Al₂O₃ composites owing to its strength, fatigue resistance, and lightweight properties, rendering it appropriate for applications necessitating structural integrity and performance. The use of Al₂O₃ particles substantially enhances hardness and wear resistance characteristics. It is lighter than steel or other high-strength materials, which are typically desired in applications where weight must be minimized.

The Al₂O₃ reinforcement particles are selected for Al7075–Al₂O₃ composites as they possess better mechanical and thermal, tribological characteristics. The addition of Al₂O₃ particles improves Al7075 matrix’s tensile strength by hindering dislocation motion. The high melting point and superior thermal stability of Al₂O₃ ensure high-temperature retention of strength in the composite. Due to its lower density on a mass basis when contrasted to other ceramic reinforcements, like silicon carbide, aluminium oxide serves as an appropriate choice for lightweight applications without sacrificing strength. Al₂O₃ is widely available and relatively inexpensive volume-adding reinforcement, compared to other ceramic additions such as SiC or B₄C.

The following section present some of the analysis conducted in the literature review to identify work on the various aluminium alloy composites. In addition to this, it presents the previous studies on the utilization of Taguchi method for optimizing the wear factors.

Nagesh D et al. investigated the wear behavior of Al6061 + Graphite + Boron composites. Al6061-Boron intermetallic, Al6061-Graphite and Al6061 + Gr + B MMC’s were successfully manufactured through the stir casting methodology. According to the findings of the investigation, the wear rate of the sample was considerably impacted by the load^11^. P Muthu et al. investigated wear characteristics of Al6061-titanium carbide-basalt hybrid MMC’s. The ANOVA findings revealed that sliding distance was the leading variable followed by weight and sliding speed^12^. Mehmet Emin Demir et al.^13^ conducted research on the mechanical and wear characteristics of AA7075-B_4_C MMCs. The SEM pictures showed that the probability of agglomeration in the base matrix enhanced as the B_4_C particulates ratio raised throughout the process. Despite the fact that B_4_C reinforcement has a tendency to cluster together, it had a favourable impact on the AA7075’s wear resistance.

Biranu Kumsa et al. conducted a wear investigation of MMC’s that were relied on the aluminium alloys. From the S/N ration analysis, it was understood that with the increase of silicon carbide in aluminium matrix, it increases the material strength as well as wear resistance^14^.

Timish Bhave et al. carried out an experiment which consisted of the fabrication and tribological investigation of AA6061-B_4_C hybrid MMC’s. ANOVA findings showed that, load is having the major effect on the wear^15^.The investigation of wear properties of Al6061-TiB_2_-CeO_2_ MMC’s were carried out by Sreenivasa et al. by employing Taguchi methodology. It was discovered that, the optimal combination of parameter are 2.5 percent titanium dioxide, 45 Newtons weight and 1250 m sliding distance. Furthermore, this combination resulted in the lowest COF among the MMC’s that were manufactured^16^.

Mehmet Emin Demir et al.^17^ investigated the mechanical and wear properties of Al7075–B_4_C composites. There value of hardness was substantially improved at a reinforcement ratio of 12%, which was observed when the influence of reinforcement ratio was investigated. The 12% B_4_C was shown to be the most effective in terms of wear resistance, just as it was in terms of hardness. There was an increase in the resistance to wear of the Al7075 matrix owing to the B_4_C particles.

Padmaraj et al. examined the wear characteristics of Al6061-B_4_C-Ash MMC’s. Statistical investigation results showed that applied load was affecting the wear rate tremendously^18^.

Manu Sama et al. examined the wear features of AA6082-3%BN-4%B_4_C-4%CC composites. The ANOVA findings indicated that, load exerted the maximum impact at 51.6% of the contribution accompanied by sliding distance and speed^19^. Taguchi analysis was employed for aluminium composites by Ikubanni et al. in order to get superior wear performance. The outcomes showed that, speed is the major important variable^20^. A wear examination of AA6061-Titanium boride MMC’s was carried out by Santha Rao et al.. The confirmation studies revealed that, the outcomes were within the 10% range of the value that was expected. The ANOVA showed that, the rotational speed is the main important process variable^21^.

Yahya Hişman Çelik et al.^22^ conducted a study to investigate the wear behaviour of AlSi7Mg2-SiC composite. According to the findings, the reduction in weight was brought about by raise in the load. The COF and the amount of weight lost both raised as the sliding distance was increased because of the increase. A decrease in weight loss was achieved despite the fact that the COF was enhanced owing to the increase in sliding speed. After analyzing the pictures obtained from the scanning electron microscope (SEM), it was determined that the creation of tribo-surfaces and deformation had a major impact on the decline in weight.

R. Vijaya Kumar et al. conducted a study that utilized the Taguchi methodology to examine the wear study of Al6061-Zircon composites. As per the response table, the most important factor for composites is load^23^. Pawandeep Singh et al. used Response Surface technique to investigate wear features of Al6061 related composites. According to the ANOVA findings, the crucial factor that affect wear rate is sliding speed^24^.

Mehmet Emin Demir et al.^25^ conducted study on the wear characteristics of Al7075–TiO_2_–Kaoline Composites. When the ratio of kaoline reinforcement was increased, it was discovered that the wear rate was also decreased. A reduction in the wear rate from 2.1 × 10^–3^ (mm^3^ m^-1^) to 1.5 × 10^–3^ (mm^3^ m^-1^) was achieved by incorporating 6 weight percent of kaoline reinforcement into the composite. Within the kaoline-reinforced composites, scanning electron microscopy (SEM) photographs of the kaoline-reinforced composites worn-out surface’s depicted that, the wear scars were narrower, and there were less pits and matrix debris. As a result of sintering and wear, the composite did not exhibit any cracks, as indicated by the findings of the SEM analysis.

Ravi Kuma et al. performed examination on the tribological properties of Al-Si_3_N_4_-SSP-RHA composites. Regression analysis was also performed and concluded that there is a high impact of speed followed by load and reinforcement on the individual wear rate^26^. Narinder Kaushik et al. performed the investigation on the wear behavior of the AMMC’s employing Taguchi approach. The outcomes of the ANOVA indicates that, the process parameters, i.e., sliding distance and SiC weight percentage have major impact on reduction of wear rate^27^.

A. Baradeswaran et al.^28^ conducted research on the mechanical and wear properties of Al7075–Al_2_O_3_–graphite composites. Furthermore, it was discovered that the incorporation of Al_2_O_3_ and Gr particulates leads to an improvement in the composite’s hardness. The hardness of Al7075–Al_2_O_3_–Gr composite was more than the Al7075 matrix. The Al7075–Al_2_O_3_–Gr composites showed excellent wear-resistance properties.

The research work carried out by B. Manjunatha et al., utilizes the Taguchi approach to examine the impact of quantity of boron carbide particulates on the Al6061 based MMC’s wear rate. From the findings, it was found that speed is the factor which has great impact on composites wear^29^. The Wear characteristics of Al6082-BN-B_4_C-Corn Cob Ash composite were investigated by Manu Sam et al. From the ANOVA results, it is found that, load is the major impacting variable representing 51.6% of all^30^.

The literature survey was conducted in order to examine earlier research on the creation of stir-casting-based aluminium alloy particle reinforced composites. An additional purpose of this literature review was to investigate the distribution of reinforcement particles with the use of a SEM apparatus. The literature review was also carried out to perform wear study of the aluminium alloy particulate reinforced composites. It is also carried out in order to investigate the optimization of the wear process variables comprising of load, speed, and sliding distance, by employing the Taguchi methodology for aluminium alloy particle reinforced composites. It is performed to identify the variable which is having greatest influence on the wear rate.

The preceding literature study indicates that numerous researchers have focused on the aluminum alloy-based metal matrix composites; however, none have investigated the manufacturing and study of properties of Al7075–Al_2_O_3_ composites. The use of Taguchi methodology to optimize the variables affecting the wear of composites consisting of Al7075 and Al_2_O_3_ is presently in the initial stages. The research on wear features of Al7075–Al_2_O_3_ composites for optimization of process parameters using Taguchi approach has also tried to improve the life and efficiency of these composites in engineering applications. This study therefore assists industries in selecting an appropriate process condition for preparing wear-resistant of Al7075–Al₂O₃ composites. It also helps industries to cut down on material degradation, enhance durability, and lower production costs.

The manufacturing and microstructural study of Al7075–Al_2_O_3_ composites are the primary focus of the current investigation. In addition to this, this research uses Taguchi method for optimizing the wear variables of Al7075–Al_2_O_3_ composites.

## Materials and details of experiment

### Materials

The high strength aluminium alloy known as Al7075 is a member of the 7xxx series and is largely alloyed with zinc. It is widely utilized in crucial sectors where strength is of the utmost importance. The Al7075 alloy has a density of 2.81 g/cm^3^. This is used in aircraft structural parts such as wings and fuselage structures and landing gears. It is also used in certain engine components, other suspension parts and wheels where strength and lightweight traits are needed. Because of its strong and light weight properties, it is used in components for satellites and spacecraft.

Alumina, also known as aluminium oxide, is a versatile substance that possesses a variety of characteristics that make it attractive for use in different applications. The size of Al₂O₃ particle used in this study is 40 microns. Its hardness, thermal stability, and electrical insulation characteristics make it ideal for use in abrasives, ceramics, electronics, and catalysis. It is also used for making aluminium metal. Its production is a critical step in many industrial and technological productions. It has a chemical formula of Al_2_O_3_. It is commonly found in white ashy solid and bulk forms, as well as crystalline shapes like corundum. Its melting point is roughly 2072 degree centigrade. Because of its excellent hardness, alumina is used in various abrasives, including grinding wheels, sandpapers, and polishing compounds. Alumina is used to manufacture high-performance ceramic materials for applications such as wear-resistant parts, electrical insulators, and refractory materials.

The plan of experiments consists of the following steps.

- Review of Literature
- Objectives Formulation
- Purchase of raw materials like Al7075 and Aluminium oxide particles.
- Production of Al7075–Al_2_O_3_ composite using Stir casting
- Microstructure analysis using SEM
- Wear study of Al7075–Al_2_O_3_ composites
- Optimization of wear parameters using Taguchi technique
- Worn-out surface analysis of Al7075–Al_2_O_3_ composite using SEM
- Results and Discussions
- Conclusions
- References

### Fabrication of Al7075–Al_2_O_3_ particulate composites

**Fig. 1:**
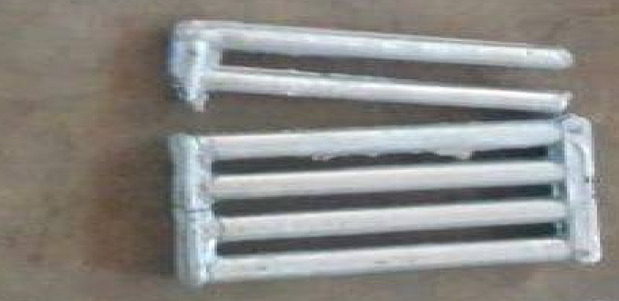
Casted Al7075–Al_2_O_3_ composite.

Composites made of Al7075 and Al_2_O_3_ particles were manufactured by employing the stir casting methodology. The matrix Al7075 alloy was in the form of ingots. The Al7075 ingots were cleaned and melted in a graphite crucible at 750 °C. In order to extract dissolved gases from the molten liquid, the solid form of hexachloroethane (C_2_Cl_6_) was utilized^31^. The overall weight was estimated by computing the weight of Al_2_O_3_ particulates at a concentration of 6% weight. The aluminium oxide particulates were heated to 300 degrees Celsius for duration of three hours to mitigate any potential thermal disparity that could arise in the casting procedure. In addition, the removal of moisture from the materials is facilitated by this preheating process^32^. A continuous stirring process was used to combine the Al_2_O_3_ particles that had been warmed with the liquid matrix. The stirring procedure is carried out for ten minutes, and the stirrer is turned at 250 rpm speed to produce a reasonable vortex, which assists in generating a uniform dispersion. A coating made of ceramic was put to the stirrer blades^33^. This was done in order to limit the amount of iron that was absorbed by the liquid metal from the drums. The molten material was put into the metal mould once the melt had been stabilized, and each of the castings can be created according to the requirements that have been specified. The stirrer was preheated at a low temperature in order to get it ready for insertion into the liquid melt before the moulds were heated to 200 degrees Celsius. This was done before the melt was poured into the moulds^34^. As a result, Al7075–Al_2_O_3_ composites were prepared. The cast samples were in cylindrical shape with a diameter and length of 24 mm and 140 mm respectively. The Al7075–Al_2_O_3_ casted composite is shown in Fig. 1.

### Wear study

The Pin on Disc instrument is utilized to carry out wear experiments on the composites composed of Al7075 and Al_2_O_3_ particles. The wear test was found to have been conducted according to the ASTM G99-95 standard^34^. The cast samples were then machined in order to acquire samples that had a length of thirty millimetres and a diameter of eight millimetres. For the wear test, a range of speeds, loads, and sliding distances were used^35^. Wear studies of Al7075–6%Al_2_O_3_ composites were conducted and a total of 27 tests were executed using Taguchi technique. For each test, three samples were tested, and an average reading was taken.

6% Al_2_O_3_ reinforcement was chosen and kept constant for the wear study of Al7075–Al_2_O_3_ composites employing Taguchi methodology. In this study, Al7075–Al_2_O_3_ composites were developed with various proportions of Al_2_O_3_. The Al_2_O_3_ particles were altered at concentrations of 2%, 4%, 6%, and 8%. The hardness test was conducted on Al7075–Al_2_O_3_ composites with various proportions of Al_2_O_3_ utilizing a Brinell hardness testing apparatus. The hardness test indicated that hardness improved up to 6% of Al_2_O_3_, after which it declined. The peak hardness is achieved at 6% Al_2_O_3_, measuring 92 BHN.

It is well known that wear and hardness are directly correlated. In order to optimize the wear variables of Al7075–6%Al_2_O_3_ composites employing Taguchi technique, 6%Al_2_O_3_ was selected and maintained constant depending on the outcomes of the hardness test of the composites. Here, the percentage of Al_2_O_3_ remained fixed while the wear process parameters such as load, speed, and sliding distance were changed in three levels.

**Fig. 2:**
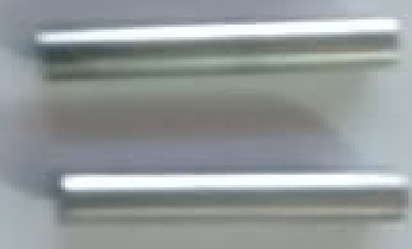
Wear test specimens.

Figure 2 shows the specimens that were used for the wear test.

Table 1 presents the wear test parameters used in the wear test.

**Table 1:** Wear test parameters.

Material of Pin	Al7075–Al_2_O_3_
Material of Disc	EN 32 Steel
Diameter of Track	114 mm
Load	10–30 (N) in steps of 10N
Sliding Speed	100–300 (m/s) in steps of 100 m/s
Temperature	Room Temperature
Sliding Distance	1000–3000 in steps of 1000 m/s

The wear rate which was calculated in the wear tests is based on weight loss. It is calculated by taking the difference between the sample weight after the wear experiment and sample weight before the wear experiment. The wear rate was calculated in the wear tests using Eq. (1).

1 $${\text{Wear}}\;{\text{Rate}} = {\text{Weight}}\;{\text{Loss/}}\left( {{\text{Density }} \times {\text{ Sliding}}\;{\text{Distance}}} \right) = {\text{W}}_{{2}} - {\text{W}}_{{1}} {/}\left( {\rho \, \times {\text{ D}}} \right)\; \left( {{\text{mm}}^{{3}} {\text{/m}}} \right)$$

The experimental density value was taken for the determination of wear rate.

### Taguchi methodology for the wear study of Al7075–Al_2_O_3_ composites

Taguchi technique was utilized for optimizing the wear performance characteristics. It is carried out by adjusting the design parameters in an appropriate manner^36^. Minitab software was utilized to optimize the variables of wear. The design variables and the levels that correspond to them are outlined in Table 2. By selecting three levels, it is possible to investigate the impacts of nonlinearity. The response variable that was investigated is the wear.

**Table 2:** Factors and levels.

Design factors	Levels		
	1	2	3
Load (N)	10	20	30
Speed (rpm)	100	200	300
Sliding distance (m)	1000	2000	3000

27 experiments were performed in order to evaluate the impact that various parameters have on the wear examination of Al7075–Al_2_O_3_ composites. Table 3 provides findings of these testes. The experimental data was analyzed using Minitab 16 software. The average values of the output values of three samples were taken for the wear test.

**Table 3:** Experimental design.

Serial number	Load	Speed	Sliding distance
1	10	100	1000
2	10	100	2000
3	10	100	3000
4	10	200	1000
5	10	200	2000
6	10	200	3000
7	10	300	1000
8	10	300	2000
9	10	300	3000
10	20	100	1000
11	20	100	2000
12	20	100	3000
13	20	200	1000
14	20	200	2000
15	20	200	3000
16	20	300	1000
17	20	300	2000
18	20	300	3000
19	30	100	1000
20	30	100	2000
21	30	100	3000
22	30	200	1000
23	30	200	2000
24	30	200	3000
25	30	300	1000
26	30	300	2000
27	30	300	3000

## Results and discussions

### Microstructure study of Al7075–Al_2_O_3_ composite

**Fig. 3:**
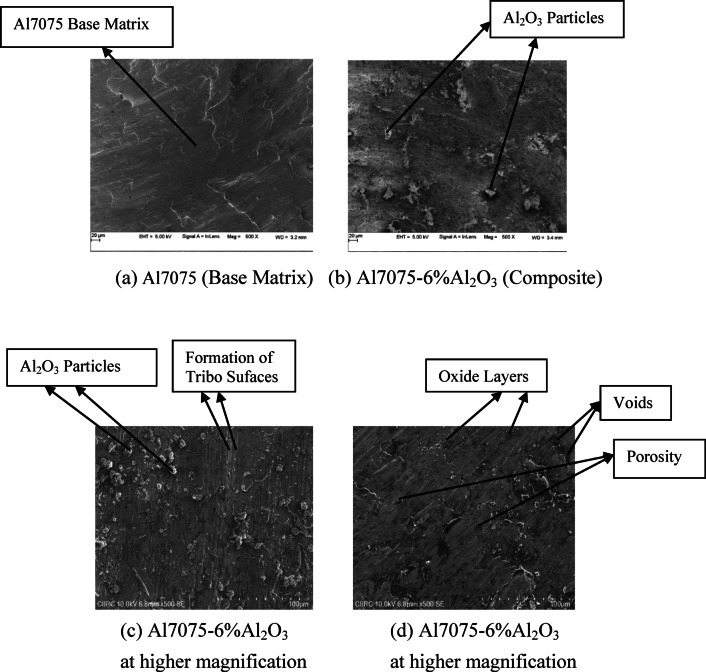
SEM picture of Al7075 matrix and Al7075–6%Al_2_O_3_ composite.

The SEM micrograph of the Al7075 matrix is shown in Fig. 3a. The SEM image of the composite material Al7075–6%Al_2_O_3_ is shown in Fig. 3b. Figure 3c,d show the SEM micrographs of the Al7075–6%Al2O3 composite material at a greater magnification.

The microstructure of the Al7075 matrix and Al7075–6%Al_2_O_3_ composite was investigated with the use of a scanning electron microscope. The Al7075–6%Al_2_O_3_ composite sample and the aluminium matrix were subjected to a particle dispersion research. According to the findings of the microstructure examination, the Al_2_O_3_ particles are distributed evenly across the Al7075 alloy matrix^37,38^. Additionally, these features existed along with the particle reinforcement. The photographs unequivocally illustrate that the Al_2_O_3_ particles have a shape that is not consistent through their entirety. The results of the microscopic study suggest that the size of the Al_2_O_3_ particles across the composite is very consistent.

### Study of worn-out surfaces of Al7075–Al_2_O_3_ composites

**Fig. 4:**
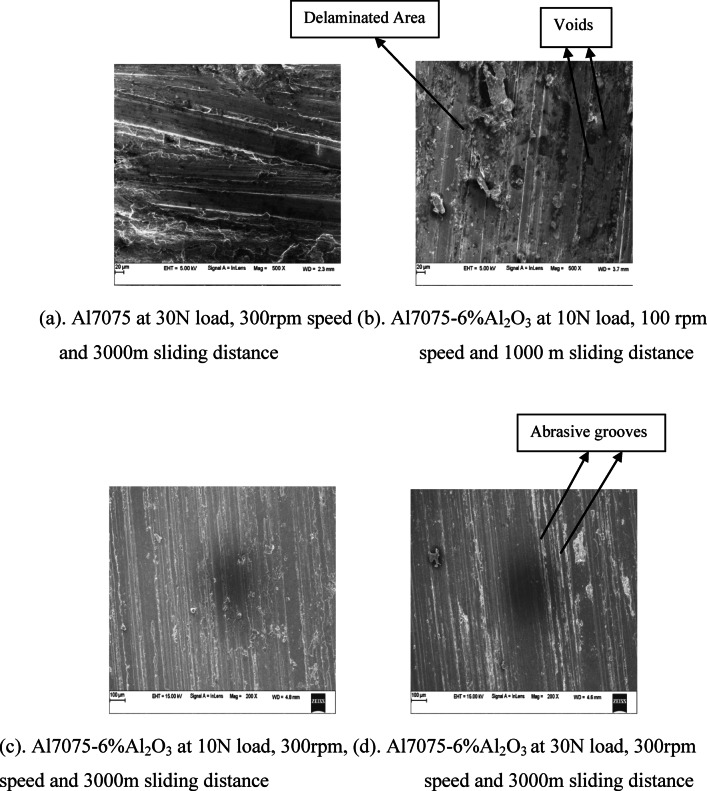
SEM pictures of Al7075 matrix and Al7075–6%Al_2_O_3_ composite’s worn-out surfaces at various loads and speeds.

The SEM pictures of Al7075 matrix and Al7075–6%Al_2_O_3_ composites worn-out surfaces are depicted in Fig. 4a–d, correspondingly. SEM pictures of the worn-out surface of Al7075–6%Al_2_O_3_ composites are displayed in Fig. 4b. These images were taken when the wear variables, which include load, speed, and sliding distance, were set to 10N, 100 rpm, and 1000 m, correspondingly. Figure 4c depicts the scanning electron microscopic pictures of the Al7075–6%Al_2_O_3_ composite’s worn-out surface when the wear variables like load, speed, and sliding distance were 10N, 300 rpm, and 3000 m correspondingly. SEM pictures of the Al7075–6%Al_2_O_3_ composite’s worn-out surface are displayed in Fig. 4d when the wear variables, which include load, speed, and sliding distance, are set to 30N, 300 rpm, and 3000 m, correspondingly.

The presence of white particulates in the images obtained using SEM is a technique that may be utilized to ascertain whether or not carbide particulates are present in the material^39,40^. As can be seen in the images, the existence of reinforcement contributed to a reduction in the width of the grooves. There are signs of delamination, which can be attributed to the occurrence, along with the formation of wear debris, which is also visible. When the composites were subjected to a wear examination, it was observed that their sliding path had grooves that were narrow and tiny, and they included a negligible amount of debris^41^. SEM images of Al7075–Al₂O₃ composites showed various wear mechanisms depending on load, sliding speed, as well as environmental condition. The SEM images show long parallel grooves in the sliding direction. Strong adhesion causes formation of wear debris. Visible sub-surface cracks or voids lead to material delamination. Large and thin wear debris particles were seen indicating brittle fracture. Isolated Al₂O₃ fragments are dispersed within the matrix^42^. The tough ceramic particles strengthen the soft aluminum, preventing deep material loss but resulting in more micro-cutting wear. Al₂O₃ particles presence limits metal-to-metal contact however, at high loads, material transfer may occur. Al₂O₃ like particles are helpful to growth of protective oxide film and wear only happened in mild state but brittle destruction occurred in large load. The delamination and wear debris decreases at higher speeds and higher sliding distances. Hence at higher sliding distances and higher speeds, the wear rate also decreases. At higher loads, the delamination and wear debris increases. Hence at higher loads the wear rate also increases^43^.

Furthermore, the insertion of strong Al_2_O_3_ particles lessens the groove width as well as the debris quantity that was formed. This, in turn, led to a reduction in the wear rates that were eventually experienced. As a consequence of the composite material’s improved load-bearing capacities, the material’s abrasion resistance has also improved, which has led to a reduction in the pace at which it wears out.

### Wear rate as a response of wear for Al7075–Al_2_O_3_ composites

An investigation on the influence that several variables have on the tribological features of Al7075–Al_2_O_3_ composites were performed through a total of 27 tests and are indicated in Table 4^44^. The average values of the output values of three samples were taken for the wear test.

**Table 4:** DOE of L_27_ OA and wear rate results.

Serial number	Load	Speed	Sliding distance	Wear rate × 10^−3^ (mm^3^/m)
1	10	100	1000	3.6131
2	10	100	2000	1.7335
3	10	100	3000	1.0583
4	10	200	1000	3.5036
5	10	200	2000	1.4233
6	10	200	3000	1.0340
7	10	300	1000	2.4817
8	10	300	2000	1.2591
9	10	300	3000	0.6812
10	20	100	1000	3.5401
11	20	100	2000	1.7153
12	20	100	3000	0.8637
13	20	200	1000	3.5766
14	20	200	2000	1.3868
15	20	200	3000	0.8759
16	20	300	1000	2.5182
17	20	300	2000	1.3321
18	20	300	3000	0.9002
19	30	100	1000	3.3941
20	30	100	2000	1.7153
21	30	100	3000	0.9367
22	30	200	1000	3.3576
23	30	200	2000	1.3868
24	30	200	3000	0.9124
25	30	300	1000	1.9708
26	30	300	2000	1.0401
27	30	300	3000	0.7664

The above table illustrates the outcomes of the wear rate response for composites made of Al7075 and Al_2_O_3_ particles when employing a L27 orthogonal array.

Table 5 provides the findings of ANOVA. This revealed that, among all the factors adhesive wear test parameters like sliding distance is the major impacting factor^45^. The presence of Al_2_O_3_ ceramic reinforcement in Al7075–6%Al_2_O_3_ composites is responsible for the lower influence of load on wear rate in comparison to sliding distance. This reduced influence could be attributed to the ceramic reinforcement’s role in moderating the wear behaviour. Despite the fact that load typically causes an increase in wear as a result of higher contact pressure, the Al_2_O_3_ particles have the ability to function as wear-resistant elements, which effectively reduces the total wear rate^46^. It is possible that this effect will obscure the direct influence that load has on the rate of wear, particularly when the load is smaller.

**Table 5:** ANOVA results.

Sources	Degree of Freedom	Sequential sums of squares	Adj. SS	Adj. MS	F	P	% Contribution	Inference
Load	2	0.1195	0.1195	0.0597	0.81	0.457	0.4350	Insignificant
Speed	2	1.9682	1.9682	0.9841	13.42	0.000	7.165	Significant
Sliding Distance	2	23.9121	23.9121	11.9561	162.99	0.000	87.057	Significant
Error	20	1.4671	1.4671	0.0734			5.3413	
Total	26	27.4669	27.4669				100	

The Al7075 matrix’s wear resistance is substantially influenced by the Al_2_O_3_ particles that are present in the matrix. During sliding contact, these hard particles have the ability to block or minimize the removal of material, which ultimately yields in a decline in the rate of wear^47^. Although load is having direct influence on wear, the overall wear resistance of the composite is also affected by other factors, such as the presence of Al_2_O_3_ particles and how they behave.

Compared to load, sliding distance has a more substantial impact since it directly determines the number of cycles or contact events that contribute to material removal. This means that sliding distance is primarily responsible for determining the cumulative wear over time.

**Fig. 5:**
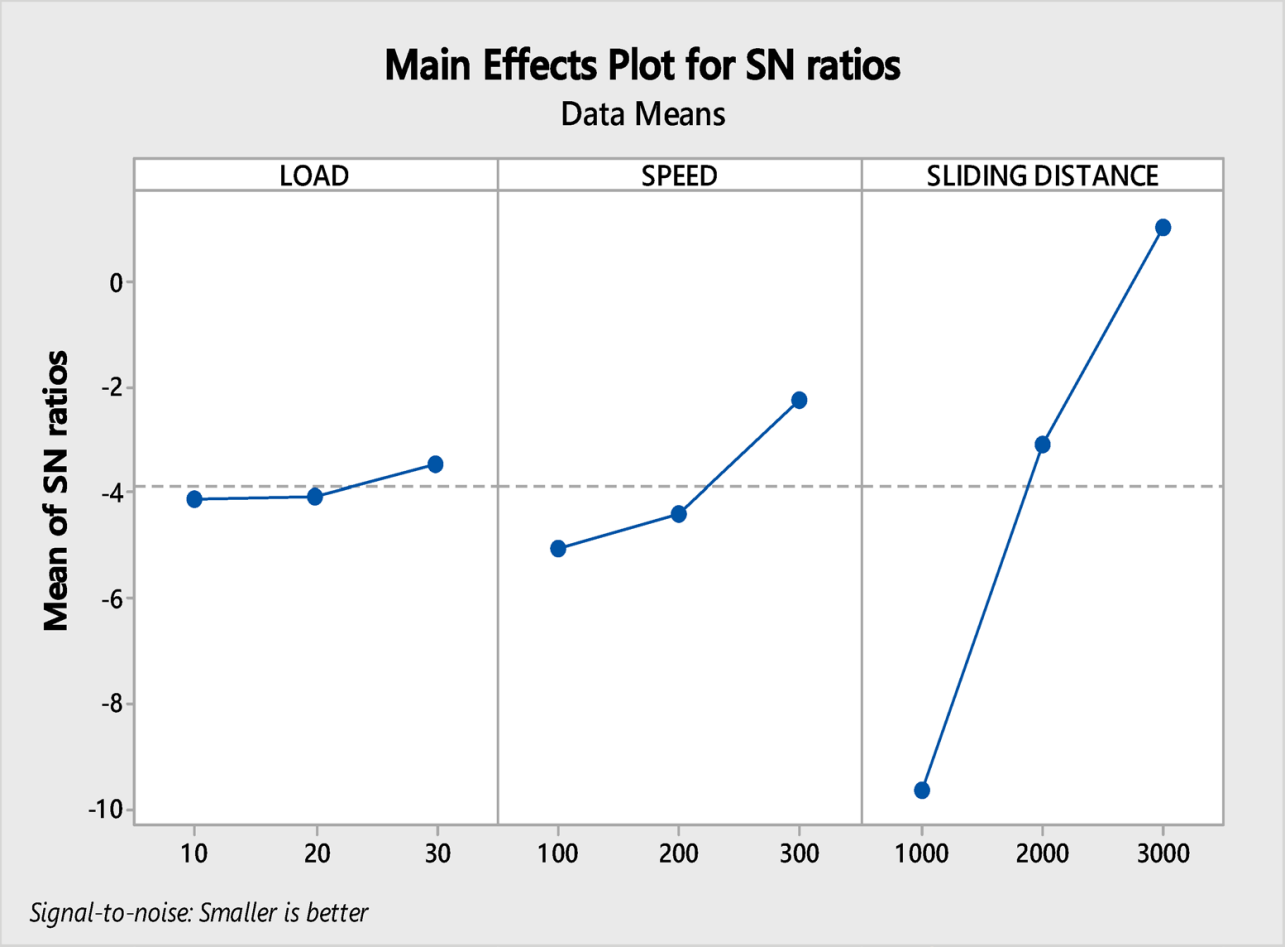
Main effect plot.

Based on the ANOVA findings, it was found that the sliding distance is the most important variable, accounting for 87.057% followed by speed 7.165% and load 0.435%. Similar results were attained by P Muthu et al.^6^. Consequently, this variable exemplifies the remarkable enhancement of the outcomes^48^. The main effect plot (MEP) is depicted in Fig. 5. It is the principal effect that has a direct influence on the variables of the response and the variable that is the dependent.

As shown in the above figure, the wear rate increases in a manner that correlates to the load, speed, and the sliding distance. Regarding the relationship among the speed, load, and the sliding distance, it was discovered that the best average response value for wear rate is level-1^49^. The wear rate diminishes with increasing sliding distance owing to the development of oxide layers, work hardening, compaction of wear debris, and surface smoothing. The wear debris, which consists of Aluminium and Al₂O₃ particles, can become compressed into the wear track by increasing sliding distance, and subsequently create an effective tribo-layer^50,51^. This layer helps to prevent metal-to-metal contact, thus limiting additional wear. With consistent sliding, the surface asperities disappear and a very smooth surface is created with extremely low friction and low rate of wear.

The wear rate lowers with the rise in sliding speed owing to increased oxide development, less adhesion, transition to mild wear, and the development of a protective tribo-layer. This causes increasing frictional heat at deeper sliding speeds, which will accelerate the generation of the Al₂O₃ oxide layer^52^. A thicker and more stable oxide layer acts as a protective film, minimizes the extent of metal-to-metal contact, thus reducing the wearing rate. At high speeds, the contact time is short, preventing the amount of sticking and transfer. At high speeds, the wear debris consisting of particles of aluminum and Al₂O₃ is compressed to form a tribo-layer. This surface layer is a full solid lubricant that lowers the friction and wear rates^17^.

**Fig. 6:**
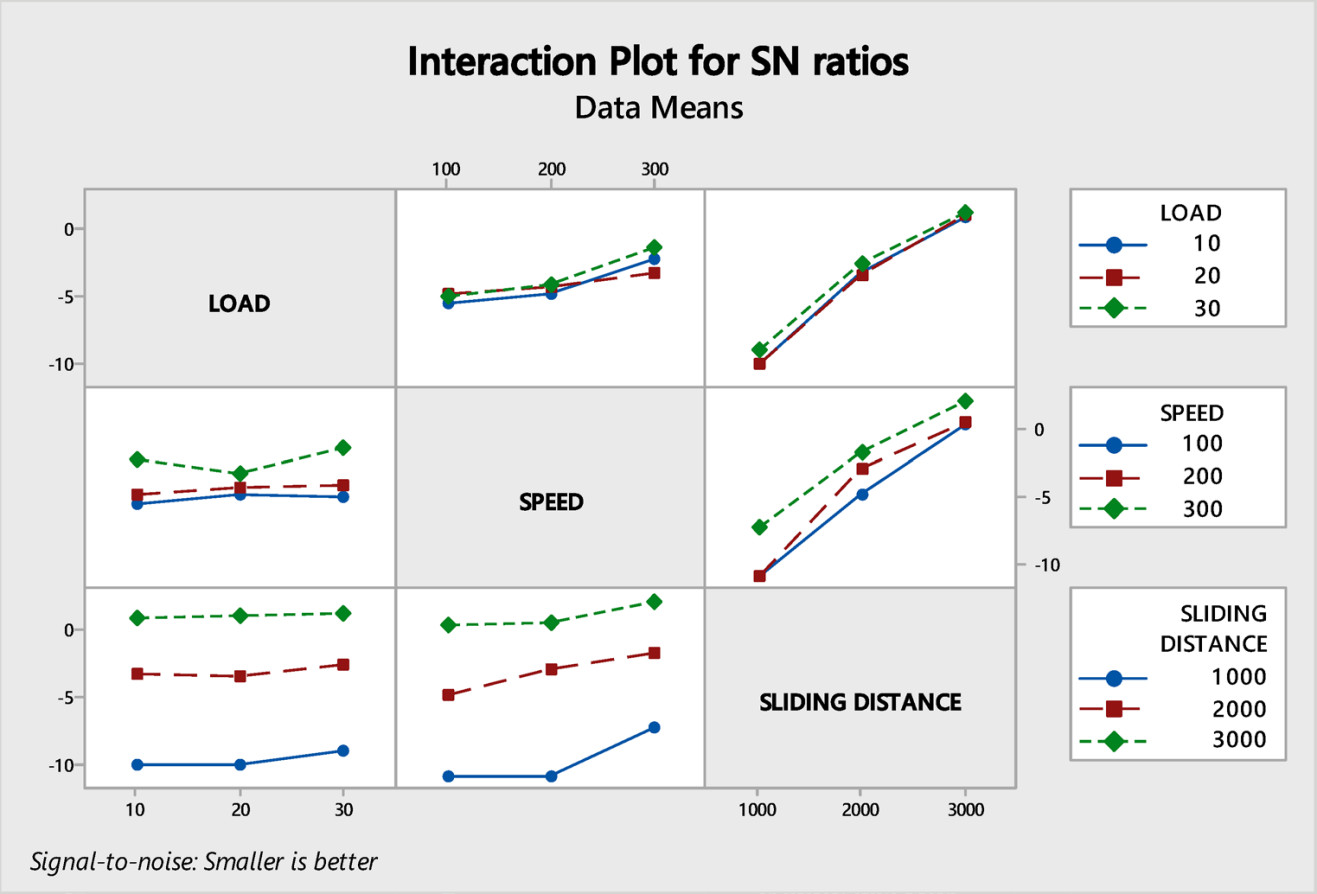
Interaction plot.

Figure 6 depicts the interaction effect that occurs between several factors and the wear rate. Upon examination of the interaction plot presented below, it becomes evident that the influence of each variable on the rate of wear is more pronounced, while the interaction between the two variables lessens the impact on wear rate.

Table 6 reveals the delta value. It has been determined that, the sliding distance is found to be the main impacting factor in terms of the delta value for wear rate.

**Table 6:** Values of delta.

Level	Load	Speed	Sliding distance
1	− 4.137	− 5.062	− 9.671
2	− 4.084	− 4.397	− 3.081
3	− 3.469	− 2.232	1.063
Delta	0.668	2.830	10.734
Rank	3	2	1

The table above illustrates the correlation between S/N ratio and the wear rate. The delta value for load, speed and sliding distance are 0.668, 2.830 and 10.734 respectively. Based on this information, it is found that, the sliding distance is found to be the main impacting factor which affects the wear rate.

#### Regression analysis

In order to generate the regression equation depending on the significance of individual factors, analysis of variance (ANOVA) is utilized. An illustration of the regression equation for wear rate can be found in Eq. 2.

2 $${\text{Wear}}\;{\text{Rate}} \times {1}0^{{ - {3}}} = {4}.{798} - 0.00{726} \times {\text{Load}} - 0.00{3122} \times {\text{ Speed}} - 0.00{11}0{7} \times {\text{ Sliding}}\;{\text{Distance}}{.}$$

The R-Sq value and R-Sq (adj) obtained using Minitab 16 Taguchi software are

$${\text{R}} - {\text{Sq}} = {95}.0{5}\% \;{\text{and}}\;{\text{R}} - {\text{Sq }}\left( {{\text{adj}}} \right) = {92}.0{8}\% .$$

#### Confirmation experiment

The confirmation test represents the ultimate phase of the DOE process. Several confirmation test trials were conducted to ascertain the wear rate. To find the optimum values for the variables affecting the signal-to-noise ratio, the MEP was employed. The optimal parameters for confirmatory test are shown in Table 7.

**Table 7:** Optimum variables for confirmatory experiment.

Optimum parameters	Load	Speed	Sliding distance
Wear	10	100	1000

The results of the experiments that were conducted for the confirmatory tests are presented in.

Table 8.

**Table 8:** Confirmatory results for wear rate.

Response	OA wear rate (mm^3^/m)	Confirmatory experimental wear rate (mm^3^/m)	Error (%)
Wear	3.6131 × 10^–3^	3.3062 × 10^–3^	8.49

The OA and Confirmatory Experimental Wear rate values are 3.6131 × 10^–3^ mm^3^/m and 3.3062 × 10^–3^ mm^3^/m respectively. The discrepancy across the experimental and Taguchi analysis value is 8.49%, which falls within acceptable bounds. Similar results were attained by Santha Rao Dakarapu et al.^13^.

**Fig. 7:**
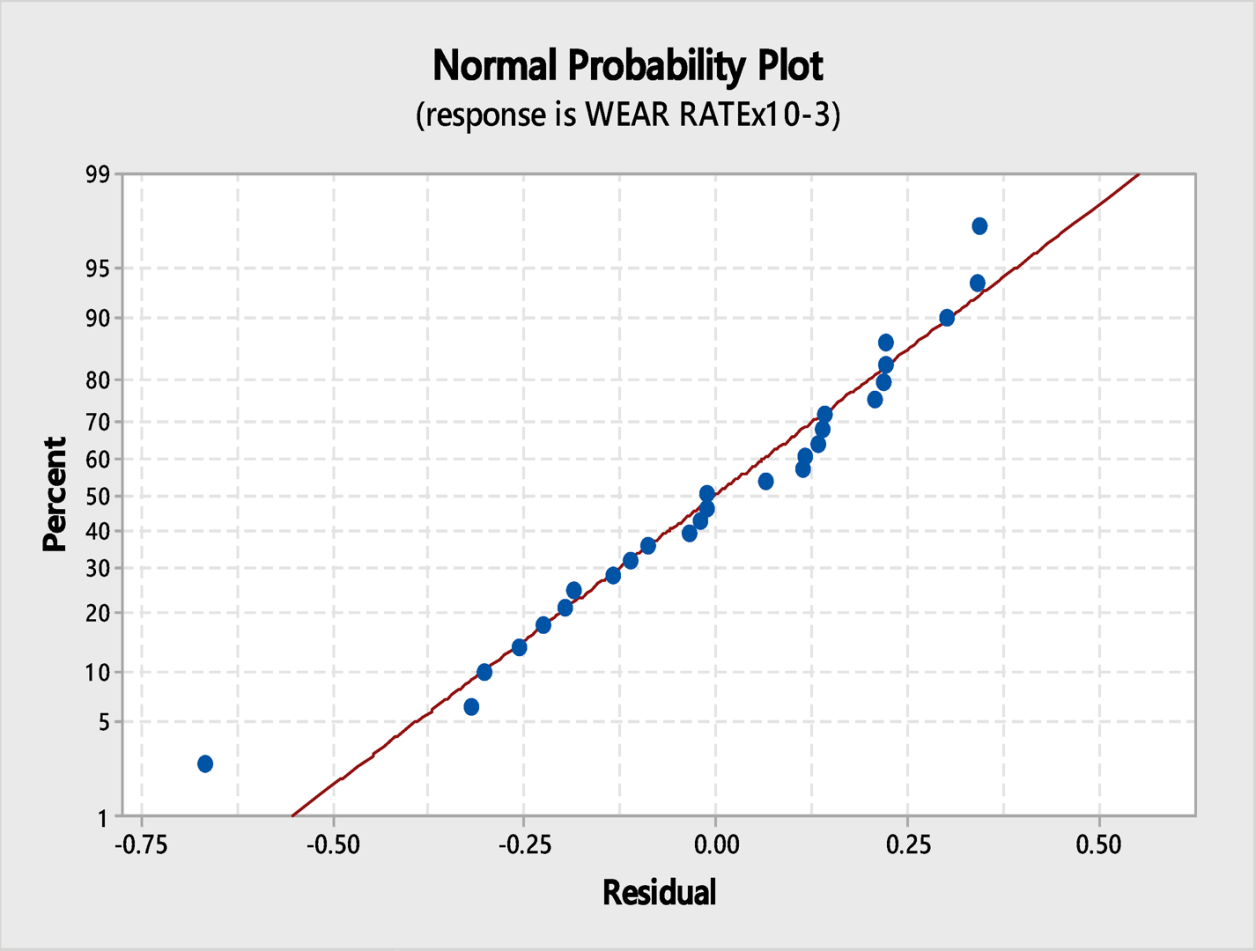
Normal probability plot.

Figure 7 is a visual representation of the normal probability plot. A closer look at this graph reveals that the spots are coming nearer and nearer to the line, which reveals that there are relatively few experimental errors.

**Fig. 8:**
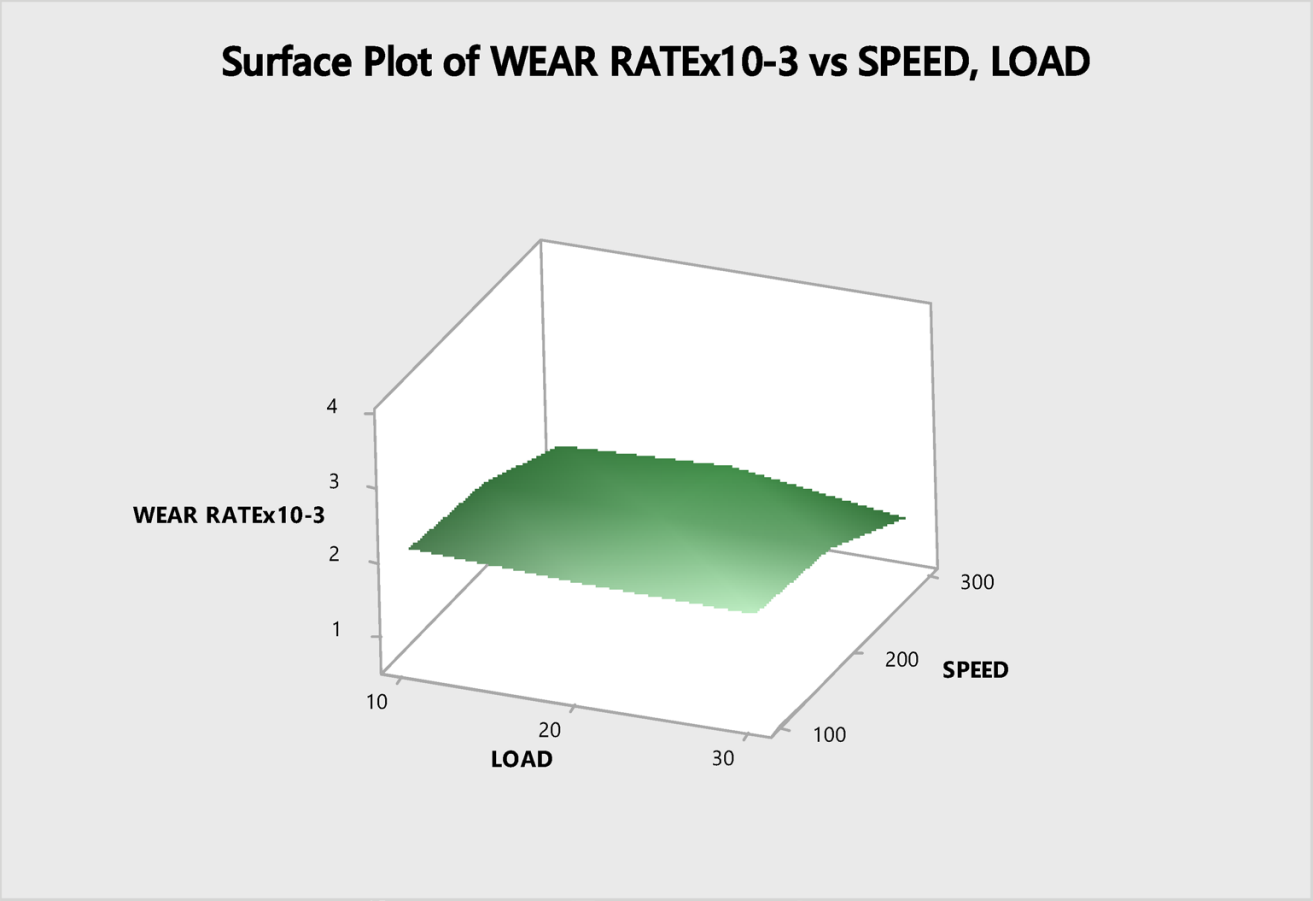
Surface plot versus load and speed.

**Fig. 9:**
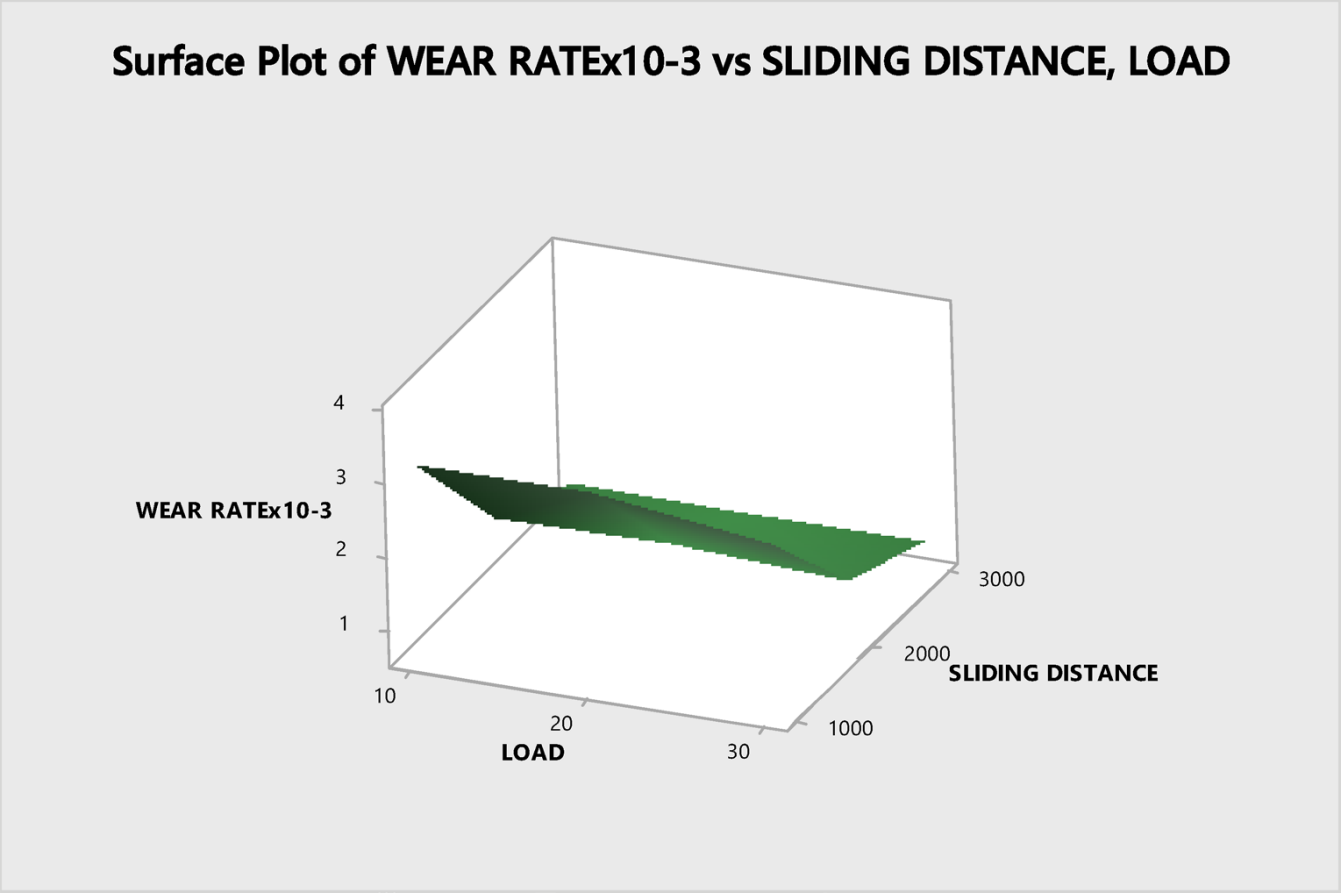
Surface plot V/S load, sliding distance.

The wear behavior was assessed by generating three-dimensional surface diagrams, which are illustrated in Figs. 8 and 9.

The Surface Plot vs. Load and Sliding distance are depicted in Fig. 8. It has been demonstrated that a high load in conjunction with a low speed is having statistically crucial impact on the rate of wear. Figure 9 illustrates how the variables like load, speed and sliding distance strongly impact the wear rate^53^. Both the increase in load as well as the sliding distance has a substantial impact on the wear rate, which is a positive relationship.

## Conclusion

This is a summary of the findings and conclusions that were obtained from the current investigation.

- The stir casting process has been utilized successfully to synthesize Al7075–6% Al_2_O_3_ composites.
- Microstructural examination of Al7075–Al_2_O_3_ composites shows that Al_2_O_3_ particles are evenly dispersed in the Al7075 matrix.
- Based on ANOVA results, it was found that the sliding distance is the most important variable, accounting for 87.057% followed by speed 7.165% and load 0.435%.
- The R-Sq value and R-Sq (adj) for wear response obtained using Minitab 16 taguchi software are 95.05% and 92.08% respectively.
- The delta value for load, speed and sliding distance are 0.668, 2.830 and 10.734 respectively. It can be deduced from this that, the crucial factor which affects the wear is sliding distance.
- The OA and Confirmatory Experimental Wear rate values are 3.6131 × 10^–3^ mm^3^/m and 3.3062 × 10^–3^ mm^3^/m respectively. The discrepancy across the experimental and Taguchi analysis value is 8.49%, which falls within acceptable bounds.
- The normal probability graph suggests that there are very few experimental errors because the spots are getting increasingly nearer to the line.
- The mean response value for wear rate is identified as level-1 concerning speed, load and sliding distance.
- The Surface Plot vs. load along with the sliding distance reveals that a crucial effect on wear rate is exerted by a combination of high load and low speed.
- The wear rate is significantly impacted by both the increase in load and the sliding distance.

## Data Availability

The data sets generated during this research are available from the, corresponding author [Subraya Krishna Bhat], upon a reasonable request.
